# Conference Report: INTERNATIONAL CONFERENCE ON MACHINING OF ADVANCED MATERIALS Gaithersburg, MD July 20-22, 1993

**DOI:** 10.6028/jres.099.009

**Published:** 1994

**Authors:** Said Jahanmir

**Affiliations:** Ceramics Division, Materials Science and Engineering Laboratory, National Institute of Standards and Technology, Gaithersburg, MD 20899-0001

## 1. Introduction

Advanced materials, such as ceramics and composites, are the building blocks of technology; but the high cost associated with their machining and finishing is a major barrier to the use of these materials in commercial applications. In some cases, current machining methods cannot be used and innovative techniques or modifications of existing methods are needed. The development of new machining technologies for advanced materials requires interdisciplinary research and collaboration between industry, government, and universities. The International Conference on Machining of Advanced Materials was held at the National Institute of Standards and Technology, Gaithersburg, Maryland on July 20–22, 1993 to strengthen communication and technology transfer among researchers and engineers involved in machining of advanced materials. This conference was cosponsored by the National Institute of Standards and Technology—Ceramics Division, the National Science Foundation—Materials Processing and Manufacturing Program, and the U.S. Navy—Manufacturing Technology Program. The conference was endorsed by the American Society of Mechanical Engineers, the American Ceramic Society, and Ceramic Industry Magazine.

The conference was attended by more than 250 people from 12 different countries representing industry, government, and universities. More than 56 percent of the attendees were from industry. A total of 64 presentations were made at the conference. A highlight of the conference was a presentation by Dr. John McTague, Vice President of Ford Motor Company, who stressed the importance of government/industry cooperation in research and technology development.

## 2. Conference Summary

To accommodate the large number of papers without having parallel sessions, the papers were divided into oral presentations and posters. In addition to the 48 papers contained in the proceedings (published as NIST Special Publication 847), 16 other presentations were made without publication. The following is a summary of the papers included in the proceedings.

### 2.1 Grinding of Technical Ceramics

The topics covered in this section included both experimental evaluation and theoretical modeling of the grinding process. A new technique, “helical scan grinding,” was described for obtaining a high degree of surface finish even with a wheel containing coarse diamond grit. The performance of single-layer diamond abrasive tools was evaluated in another paper to develop guidelines for the design and implementation of these tools for grinding of ceramics. An electro-discharge trueing operation was described for metal-bonded diamond grinding wheels to achieve a higher grinding ratio and lower grinding forces than conventional means of trueing. A new Technique, based on self-sharpening of the diamond wheels, was presented for increasing the material removal rate during grinding of ceramics. The controlling effect of grinding fluids on the material removal process was described and a new additive was proposed for improving the drilling rate of alumina ceramics by diamond abrasive drills. One of the papers emphasized the importance of a systems approach in grinding of ceramics. In this approach, the machine tool, diamond wheel, work material, operational factors, and cost of the machining cycle were examined to design cost-effective production processes for ceramic grinding. A simple model was presented relating grinding forces to the operational parameters in grinding. The basic process of grinding was modeled analytically by describing the stress field caused by sliding microindentation of brittle materials. The papers in this section presented information on grinding of alumina, silicon nitride, silicon carbide, tungsten carbide, zirconia, and alumina fiber-reinforced composites.

### 2.2 Characterization of Machined Surfaces

Nondestructive techniques were described for the characterization of surface roughness and machining-induced subsurface damage in ceramics. In one paper, a sharply focused ultrasonic transducer was used to generate short-duration pulses for the detection of subsurface damage in silicon nitride. In a second paper, the surface morphology of ultrasonically machined glass, alumina, and zirconia were evaluated by surface profiling and scanning electron microscopy to determine the material removal process. An indentation technique was used to measure the residual stresses in ground and polished soda lime glass, Ni-Zn ferrite, and silicon nitride, and the results were compared with x-ray diffraction measurements. Optical scattering methods were successfully applied to detect subsurface defects in ground and polished silicon nitride samples. As an in-process method, acoustic emission sensors were used to detect chatter and surface patterns in cylindrical grinding.

### 2.3 Effect of Grinding on Strength

The effects of grinding-induced damage on strength and surface quality of silicon nitride, alumina, and other types of ceramics and ceramic composites were reported. The effects of cutting speed, depth of cut, diamond grit size, grinding direction, ceramics grain size and microstructure on strength were evaluated in several papers. Also, the relationships between grinding parameters and residual stress were determined by x-ray diffraction. It was shown that the magnitude of compressive residual stress in silicon nitride increases as the chip thickness is increased in grinding. The bending strength of various ceramics was shown to be dependent on the grinding direction with respect to the direction of the tensile axis. The strength of samples ground in the longitudinal direction (i.e., parallel to the tensile axis) did not depend on the grinding condition, even when the removal rate was increased by a factor of 60. However, the strength of samples ground in the transverse direction (i.e., perpendicular to the tensile axis) was affected by the parameters used in grinding. The magnitude of the strength in the transverse direction was generally lower than the samples ground in the longitudinal direction. However, the reduction in strength was related to the specific grinding conditions used and the ceramic microstructure, such as the grain size. It was also reported that the specific grinding energy is a function of ceramic grain size.

### 2.4 Precision Machining

The papers in this section were focused on both single-point diamond turning and “ductile” regime precision grinding. The wear mechanisms on the diamond tools used in single-point cutting were found to be primarily controlled by solid state diffusion rather than abrasion. Micro-Raman spectroscopy was used to determine the residual stresses in germanium crystals machined by single-point turning. A new design concept for ultra-precision grinding machines with vertical spindles was proposed for “ductile” regime grinding of ceramics. Several papers described new methods for trueing and dressing of diamond wheels for the generation of precision aspheric surfaces. The electrolytic in-process dressing technique was reviewed in particular and some recent results were presented. Two papers showed that “ductile” regime grinding can improve the surface finish and strength of silicon nitride components if the machining-induced damage from rough grinding is removed.

### 2.5 Free-Abrasive Machining

Free-abrasive machining refers to the processes there material is removed by loose-abrasive partiles. The ultrasonic machining process was evaluited for machining of feldspathic porcelain (a lental ceramic material). It was found that the sending strength and fatigue resistance were improved by ultrasonic machining as compared to conventional grinding and lapping techniques. An energy-based model was proposed for cutting of carbon-reinforced polymer composites by water jet and abrasive water jet machining. Two papers were focused on new concepts for polishing. In one paper, mechanochemical polishing of silicon nitride was achieved by incorporating chromium oxide particles into the grinding wheel. In the second paper, a magnetic-abrasive finishing technique was effectively used to polish tungsten carbide.

### 2.6 Turning and Milling

Conventional techniques of turning and milling are routinely used for fabrication of metallic materials; however, their use for advanced materials is limited to “softer” ceramics, metal-matrix composites, and polymeric composites. Machining of advanced materials is often difficult and requires development of new cutting tools and machining porcedures. In one paper, the machining processes used for ceramic dental restoration materials were reviewed; and statistical design of experiments was shown to be an effective means for the optimization of the machining process with respect to the surface roughness and removal rate. In another paper, the machinability of several dental materials, such as glass-ceramics, lucite-reinforced ceramics, dental hybrid composites, and hydroxyapatite, were evaluated with respect to surface quality, Several types of aluminum-matrix composites containing ceramic reinforcements were machined by turning to select suitable cutting tool materials. It was found that deposition of a diamond film on a silicon nitride tool gives the lowest wear rate among all the tool materials and coatings tested. In a similar study, performance of different tool materials, such as cemented carbide, alumina composites, sialon, cubic boron nitride, and polycrystalline diamond, were evaluated for machining of carbon and glass fiber-reinforced phenolic resin composites. The mechanisms of tool wear in turning of fiber-reinforced polymers were studied with both plain and coated cemented carbide tools. The performance of polycrystalline cubic boron nitride tools was also reviewed for machining of hard steels. Turning was applied to alumina ceramics using a polycrystalline diamond tool. An analysis of the surface indicated that although the surface contained a few residual microcracks, the surface roughness was within an acceptable range for most applications. In one paper, a new laser-assisted, hot-machining process was proposed for turning and milling of ceramics. In this experimental process, the laser beam is used to heat and soften the material just ahead of the cutting zone to reduce the cutting forces.

### 2.7 Laser and Electrical Discharge Machining

The application of pulsed lasers in machining of advanced materials was reviewed in this section. The influence of heat-affected layers and residual stress on fracture strength of finished test samples was evaluated. It was shown that the fracture strength of laser-processed silicon nitride ceramics was lower by 10 to 20 percent compared with ground samples. The process of material removal in laser machining of silicon nitride was shown to be dissociation and oxidation of silicon nitride. The finished surfaces were found to contain porosity, an oxidized layer, and a heat-affected zone which reduced the surface quality and strength. It was reported that although laser processing of ceramics cannot be used alone to finish advanced ceramics, it can be used as a complementary process to conventional diamond grinding, especially in high-speed cutting of complicated shapes and geometries. Electrical discharge machining as applied to ceramics and polycrystalline diamond was reviewed. It was shown that several ceramics such as titanium diboride, boron carbide, and several composites containing nitrides, carbides, and borides can be cut by electrical discharge machining. Machined surfaces were examined to assess the material removal mechanisms and surface quality.

## 3. Future Conference

Based on the comments made by attendees, this conference was successful in achieving its goal, which was to strengthen communication and technology transfer among researchers and engineers involved in machining of advanced materials. Many participants suggested that the conference be held on a regular basis. Following discussions with the Conference Advisory Committee it was tentatively decided to hold the Second International Conference on Machining of Advanced Materials in Germany in 1996. If this conference is successful, the next conference will be held in Japan, Then, plans will be made to rotate the conference every 2 years between the United States, Germany, and Japan.

## 4. For More Information

Copies of the Conference Proceedings are available through the National Technical Information Service (NTIS), Springfield, VA 22161. The following information should be used when ordering this item from NTIS: Machining of Advanced Materials, NIST/SP 847, PB93-217578/AS. Please indicate if you are ordering a hard copy (A23/$61.00) or a microfiche (A04/$19.50).

To obtain more information about the next conference, or to be placed on the mailing list, write to Said Jahanmir, National Institute of Standards and Technology, Building 223/Room A329, Gaithers-burg, MD 20899-00001.

